# Tunable Compact Metamaterial-Based Double-Negative/Near-Zero Index Resonator for 6G Terahertz Wireless Applications

**DOI:** 10.3390/ma15165608

**Published:** 2022-08-16

**Authors:** Alya Ali Musaed, Samir Salem Al-Bawri, Mohammad Tariqul Islam, Ahmed Jamal Abdullah Al-Gburi, Mandeep Jit Singh

**Affiliations:** 1 Space Science Centre, Climate Change Institute, Universiti Kebangsaan Malaysia (UKM), Bangi 43600, Malaysia; 2 Department of Electrical, Electronic and Systems Engineering, Faculty of Engineering and Built Environment, Universiti Kebangsaan Malaysia (UKM), Bangi 43600, Malaysia; 3 Center for Telecommunication Research and Innovation (CeTRI), Faculty of Electronics and Computer Engineering (FKEKK), Universiti Teknikal Malaysia Melaka (UTeM), Durian Tungal 76100, Malaysia

**Keywords:** THz, metamaterial, DNG, ENG, MNG, wireless communication, 6G

## Abstract

This paper introduces the tunability performance, concept, and analysis of a unique and miniaturized metamaterial (MTM) unit cell covering the upcoming 6G applications. The proposed metamaterial consists of two metallic star-shaped split-ring resonators (SRR). It has a line segment placed in the middle of the structure, which can feature tunable characteristics. The proposed design provides dual resonances of transmission coefficient S21 at 0.248 and 0.383 THz with a significant operating frequency span of 0.207–0.277 and 0.382–0.390 THz, respectively. Moreover, wide-range achievement, negative permittivity, double-negative (DNG) refractive index, and near-zero permeability characteristics have been exhibited in two (z and y) principal wave propagation axes. The resonance frequencies are selective and modified by adjusting the central slotted-strip line length. Furthermore, the metamaterial is constituted on a polyimide substrate while the overall dimensions are 160 × 160 μm^2^. A numerical simulation of the proposed design is executed in CST microwave studio and has been compared with advanced design software (ADS) to generate the proposed MTM’s equivalent circuit, which exhibits a similar transmission coefficient (S21).

## 1. Introduction

Integration of satellite and ground-based communication networks will mark the 6G era. The 6G frequency band is an expansion from the 5G millimeter-wave band to the terahertz range. Today’s wireless communication systems are the equivalent of the eureka moment, owing to the rapid technological developments during the last several decades. Moreover, the exponential development of sophisticated technologies such as artificial intelligence (AI), Three-dimensional (3D) media, virtual reality (VR), robotics, and the internet of everything (IoE) might alter the future of wireless communication. This expansion of wireless communication has caused a significant increase in traffic capacity [1,2]. These breakthroughs have encouraged businesses and academics to begin conceiving the sixth generation (6G) of wireless communication systems to meet the communication demands of the 2030s [3] and preserve the sustainability and competitiveness of wireless communication systems [4].

Metamaterials (MTMs) are a category of artificial structures with qualities not found in nature. It has been neither presented naturally in compounds on our planet nor in our larger interplanetary environment (at least in terms of the ones that have not yet been identified in the history of scientific research, observation, and human understanding) [5,6,7]. MTMs are considered a collection of materials with exceptional and/or unique features that materials in nature do not normally display [8,9]. Typically, MTMs are characterized (or classified) according to their scattering capabilities or constitutive properties [10,11]. Depending on the values of the dielectric constants, MTMs can be single-negative (SNG) or double-negative (DNG)/left-handed (LH), the MTM is called an SNG MTM if either the permittivity (ε) or the magnetic permeability (µ) is negative. The SNG MTM with a negative ε is called the epsilon negative (ENG) MTM, and the SNG MTM with a negative µ is called the mu-negative (MNG) MTM. Lastly, DNG/LH MTM is the name for an MTM that has negative permittivity and permeability [12,13]. Metamaterials are not new to the field of telecommunications, and their popularity has increased in recent years in several disciplines, including electromagnetics, optical communication [14], state physics, microwave and antenna engineering, material sciences, and nanoscience. Metamaterials are electromagnetic materials that have been created to meet certain specifications. These new features occur as a result of unique electromagnetic field interactions or external electrical control [15,16]. In most instances, the size, form, or geometry of a metamaterial determines its distinctive and desirable characteristics. Light can be manipulated using metamaterials, electromagnetic (EM) waves, sound, and even mechanical and electrical forces, as matter has gravitational characteristics [9,17]. They have the specificity to have a negative permeability, a negative permittivity, and a negative refractive index. They can control or modify the permittivity and permeability of the material to achieve a suitable behavior for a specific application. They are used to improve the performance of antennas, filters, and couplers [12,18,19].

Researchers have suggested remarkable designs to produce a low profile, low cost, and highly effective metamaterial. A design and fabrication of an isotropic terahertz (THz) metamaterial for dual-band operation was presented in [20]. The suggested metamaterial, which consists of a regularly arranged double metallic-ring resonator, achieves negative permittivity in the frequency ranges of 0.298–0.388 THz and 0.652–0.700 THz. On a GaAs substrate, layers of titanium, platinum, and gold, all of which are costly to fabricate, compose the metamaterial. The authors in [21] present a THz-tunable negative refractive index metamaterial based on graphene. The suggested metamaterial unit is composed of a metallic resonant structure and embedded graphene, which exhibits a dynamic negative refractive index in the frequency band of 4.245 to 4.275 THz. To examine the practical use of metamaterials, a dynamic beam-tilting antenna loaded with a metamaterial-based stacking structure has been developed, and changing the chemical potential of the embedded graphene allows for the adjustment of the steering angle. Reference [22] presented a certain circular split-ring resonator metamaterial unit cell fabricated on a quartz substrate that is used to improve antenna performance. By incorporating a metamaterial into a conventional microstrip patch antenna, the antenna’s size is reduced while its performance is greatly improved. However, the antenna exhibited just a single resonance frequency at 1.02 THz. The author in [23] explored the design and radiation parameters of a planar antenna for the frequency range of 0.34 to 0.4 THz, with a focus on 0.37 THz. The proposed antenna consists of a metasurface composed of a periodic array of square patches printed on the substrate’s top side, and a planar feeding structure, both of which are patterned on a high-permittivity GaAs substrate. A fishnet-based metamaterial (MTM) loaded rectangle microstrip patch antenna (RMPA) is presented in [24]. At a frequency range between 1.05 and 1.1 THz, using a quartz dielectric substrate and a silver conductor part silver, a negative refractive index of the MTM has been observed in the corresponding band.

In this paper, a star-shaped split-ring resonator tuned metamaterial is presented that shows several resonances of S21 covering different THz bands. The novelty of this design is that the MTM’s resonator patch can be split by a slotted-strip line at the center to perform tunning properties by adjusting its length. Moreover, this MTM provides negative permittivity, near-zero permeability, and a negative refractive index in the frequencies 0.25–0.32 THz and 0.391–0.396 THz. In addition, it operates on the *Y*-axis with negative permittivity, permeability, and refractive index.

The rest of the manuscript is organized as follows: the first will be “Unit cell metamaterial design and simulation” and will examine the design evaluation steps as well as the equivalent circuit design and simulation. The second section, “Frequency tuning of proposed MTM”, discusses the tuning property. Included in “Result and Analysis” is the investigation of metamaterial performance and characteristics, such as permittivity, permeability, refractive index, impedance, as well as the electric field, surface current, and magnetic field investigation.

## 2. Metamaterial (MTM) Unit Cell Design and Simulation

**Figure 1:**
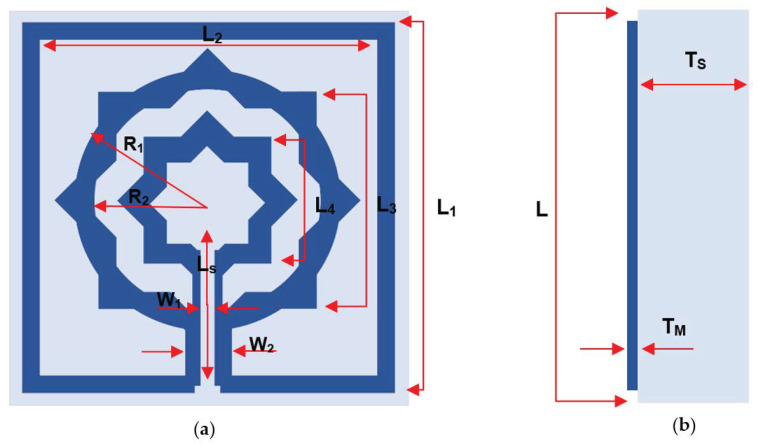
Proposed MTM unit cell: (**a**) front view; (**b**) side view.

The proposed MTM unit cell is designed on a polyimide substrate having a thickness of 50 µm, dielectric constant of 3.5, and loss tangent of 0.00027. The dimension of the substrate is selected as 160 × 160 µm^2^. The resonating patch is constructed on this substrate material with copper (Cu) as a conductor, which is 0.4 μm thick and has conductivity and resistivity values of 5.8 × 107 S/m and 1.68 × 10^−8^ Ω.m, respectively. The unit cell is composed of two star-shaped split rings with one capacitive charge in the middle. The length of this center slotted-strip line is crucial for altering the resonance frequency. The frequency of resonances may be changed by adjusting the length of this line. Figure 1a depicts the front view of the structural arrangement of the proposed unit cell, while Figure 1b demonstrates the side view. All the metamaterial parameters are illustrated in Table 1. The proposed and modified metamaterial shape may vary its transmission capacity, reflectance, and coupling. The overall unit cell configuration of a metamaterial is shown in Figure 1.

## 3. Evolution Steps of the Proposed MTM Unit Cell

**Figure 2:**
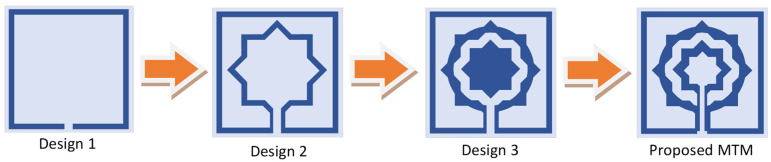
Evaluation steps toward the metamaterial design.

**Figure 3:**
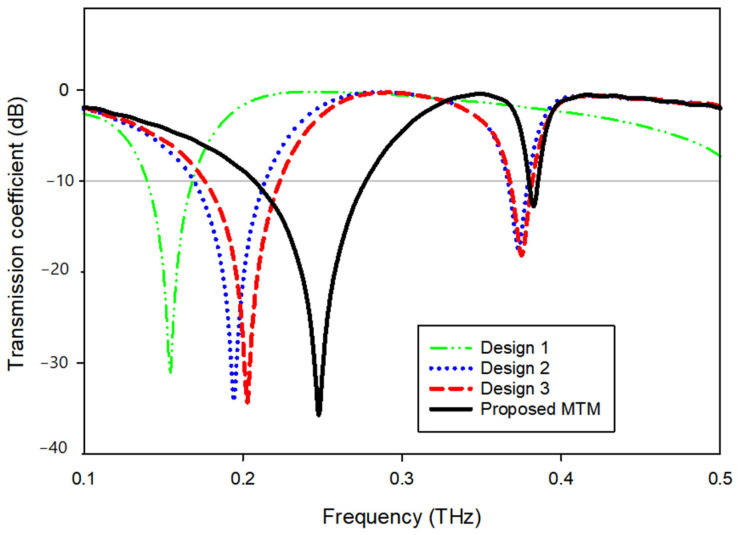
Transmission coefficient for evolution steps toward the proposed MTM.

As seen in Figure 2, the proposed MTM design is completed step-by-step and by monitoring the reaction of transmission coefficient (S21) for various design configurations, as depicted in Figure 3.

The design is initiated with a star-shaped ring with eight angles and a square frame with a slot in the bottom middle; design (1), seen in Figure 2, shows a single resonance frequency at 0.157 THz. As indicated in design (2), the ring is then cut and connected to the frame. This construction offers double resonance frequencies at 0.194 and 0.373 THz, as illustrated in Figure 3. In design (3), as depicted in Figure 2, a smaller star-shaped structure is placed in the center, and the previous ring is enhanced. Mutual inductance between the two rings modifies the inductive action of the first ring, causing a shift of earlier resonance to 0.203 and 0.375 THz, as shown in Figure 3. By expanding the slotted-strip line that links the frame and the first ring until it reaches the middle ring, the frequency shifted significantly to reach 0.248 and 0.383 THz due to the capacitive effect that is caused by the slotted-strip line. Upon observing the resonant frequency movement capability, the slotted-strip line is seen to be accountable for the frequency shifting. Now the proposed metamaterial design consists of two rings with a metal slotted-strip line that connects them together with achievable dual resonance frequencies. Table 2 summarizes the S-parameter’s resonant frequency, ranges, and peak value.

## 4. Equivalent Circuit Modeling and Simulation

**Figure 4:**
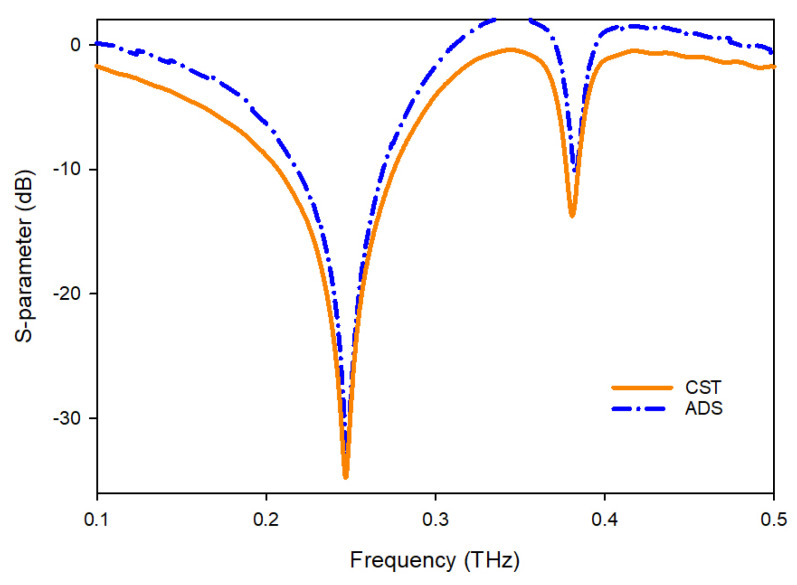
S21 comparison of the equivalent circuit result with simulation.

Numerous attempts have been made by researchers to create a model of the equivalent circuit. Due to the magnetic induction caused by current flow, it is possible to build the equivalent circuit of the proposed metamaterial unit cell by considering metallic conductors having inductor properties. Figure 4 compares the simulation results of the advanced design system (ADS) and CST to determine if a comparable circuit represents the desired unit cell. It is evident that the ADS and CST simulations provide almost identical results, although with a narrower bandwidth and greater peak resonance. The resonance frequency (*f*_0_) of the metamaterial unit cell may be calculated using the formula below [25]:

(1) $$f_{0}=\frac{1}{2\pi \sqrt{LC}}$$

Here, *L* and *C* denote the unit cell’s inductance and capacitance, respectively. The following equation [25] describes the capacitance generated by the gap in the rings, as well as the gap between the rings.

(2) $$C=\varepsilon_{0}\varepsilon_{r}\frac{A}{d}\left(F\right)$$

where $\varepsilon_{r}$ represents relative permittivity and $\varepsilon_{0}$ represents permittivity in free space. *A* represents the area of the gap, whereas *d* represents the distance of the break or gap in the metal strip or rings. The inductance of the metal strip is determined using the following equation, which is based on the transmission line concept [26]:

(3) $$L=2\times 10^{-4}l\left[\ln\left(\frac{l}{w\times t}\right)+1.193+0.2235\left(\frac{w\times t}{t}\right)\right]k_{g}$$

where *L* represents the inductance, *w* represents the microstrip line’s width, *l* represents the microstrip line’s length, and *t* represents the microstrip line’s thickness. $K_{g}=\left(0.57-0.145\ln\frac{w^{\prime }}{h^{\prime }}\right)$ here, *w*′ is the width and *h*′ is the thickness of the substrate [27].

**Figure 5:**
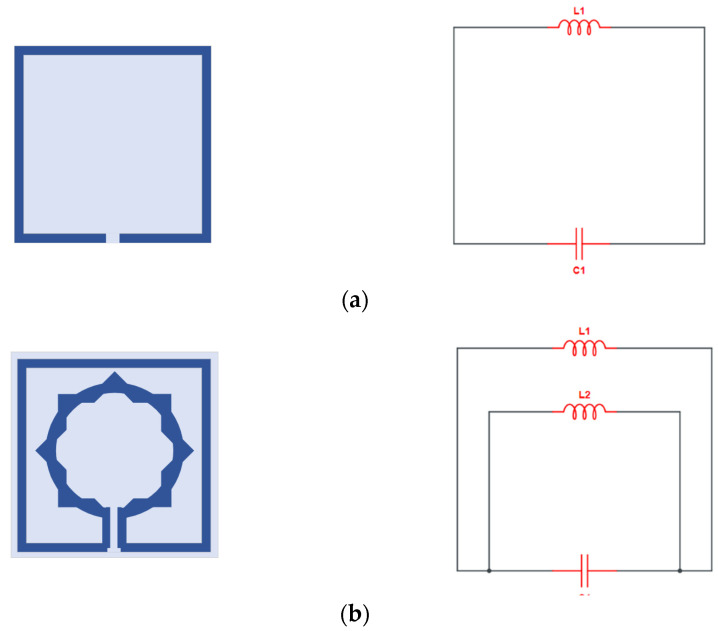

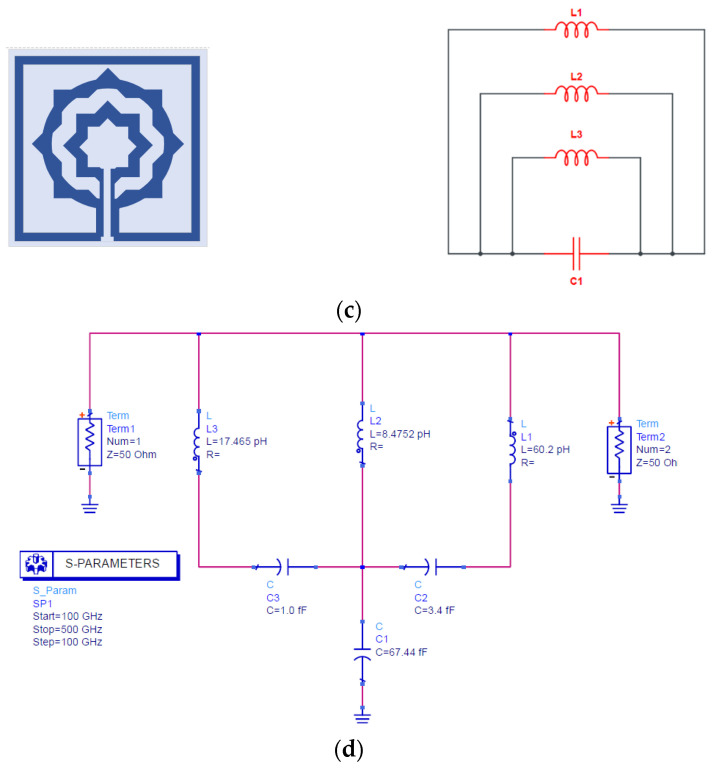
Equivalent circuit model: (**a**) single split-ring resonator; (**b**) two split-ring resonators; (**c**) three split-ring resonators; (**d**) final obtained equivalent circuit model.

While Figure 5 shows the evolution of the equivalent circuit of the proposed unit cell, note that the MTM unit cell suggested has both inductive and capacitive components. In addition, the conducting strips of the ring resonators create an inductor (L1) and split or gap in the ring, while the space between the rings constitutes the capacitor (C1) as shown in Figure 5a. Then in Figure 5b, a second split ring is added that serves as an inductor (L2) and is linked to the capacitor (C1). Figure 5c shows that the second ring is added and provides an inductive reactance (L3) link to the split performed by (C1). Therefore., this MTM produces the LC resonance circuit, demonstrating the resonances. For the sake of circuit analysis, microstrip lines are represented as multiple inductors, L1, L2, and L3, while the capacitor (produced by the split gap) is represented as C1, C2, and C3. C2 and C3 are coupling capacitors in the equivalent circuit between the resonator rings, as illustrated in Figure 5d.

## 5. Frequency Tuning of Proposed MTM

**Figure 6:**
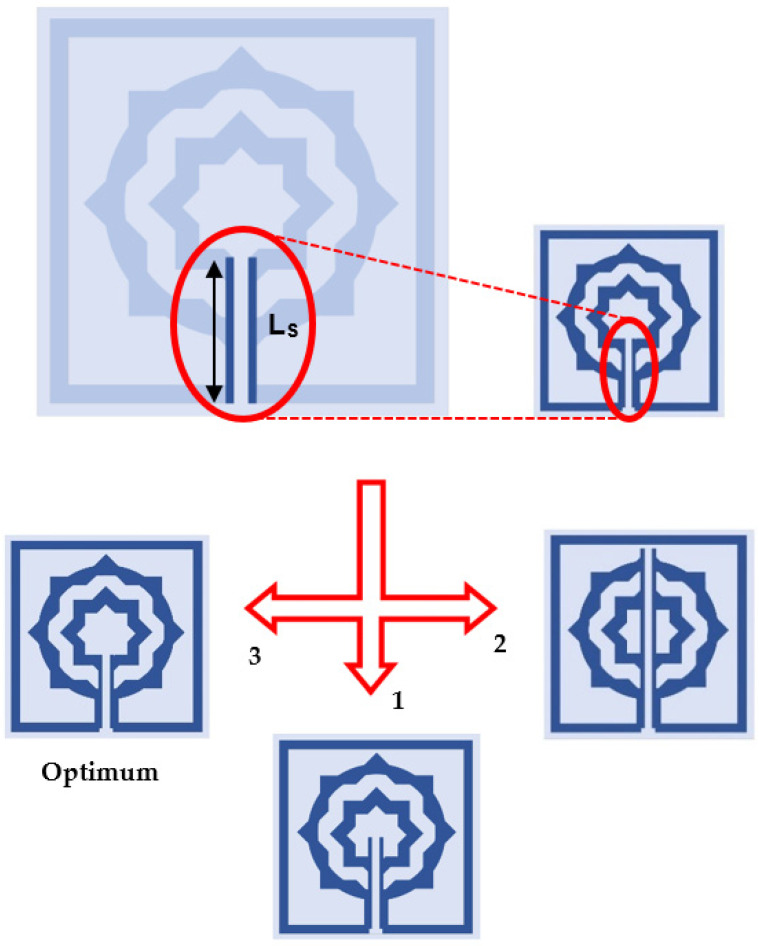
Tuning slotted-strip line of the proposed MTM.

Using a vertical strip line with a slot situated in the center, as seen in Figure 6, the scattering properties of the proposed MTM will be changed according to application requirements.

**Figure 7:**
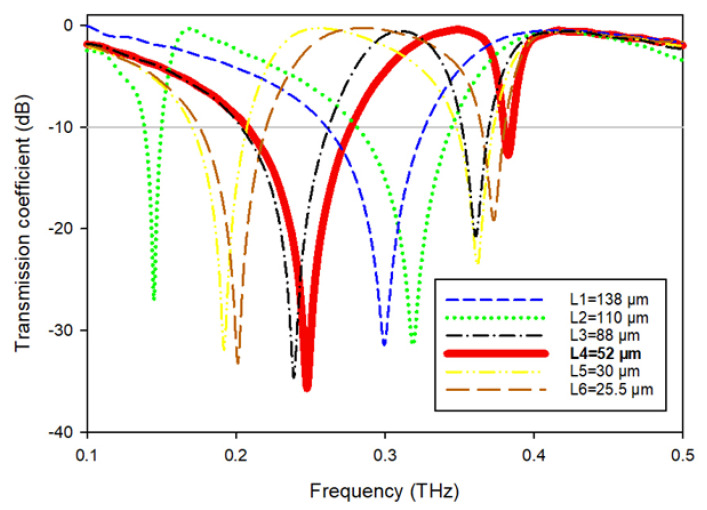
Transmission coefficients while varying the slotted-strip line’s length.

**Figure 8:**
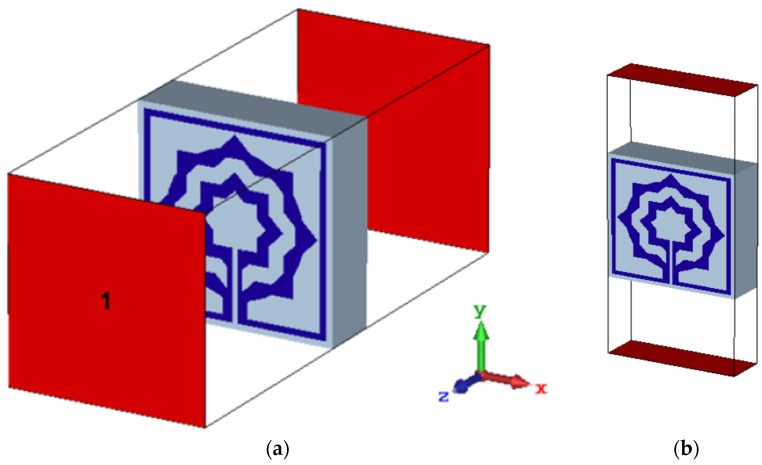
Metamaterial simulation set up: (**a**) on *Z*-axis; (**b**) on *Y*-axis.

To monitor the reaction of the MTM, the length (Ls) of this slotted-strip line is altered concurrently from its maximum length of 138 µm to its minimum length of 25.5 µm with a 4 µm wide slot. Figure 7 depicts the resonances in transmission coefficients resulting from these changes in strip line length. The maximum bandwidth is 0.067 THz with a center frequency of 0.247 THz, whereas the minimum bandwidth is 0.116 THz with resonance at 0.145 THz, assuming that a plane wave travels in the *Z*-direction and the electric and magnetic fields orient in the *X*- and *Y*-directions, respectively, as shown in Figure 8a. Table 3 summarizes the impact of altering the slotted split line on frequency and bandwidth while the wave moves along the *Z*-axis.

### Effect of Port Relocation on Tunning Property

**Figure 9:**
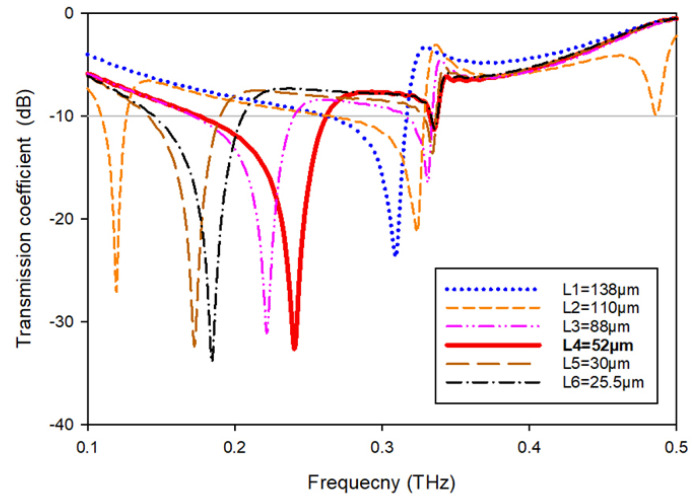
Transmission coefficients tuning when repositioning the ports in the *Y*-direction.

The findings are obtained before with the port orientated along the *Z* axis. When repositioning the ports in the *X*- and *Y*-directions, it is also required to examine the impact of various port placements on tuning properties, scattering, and effective parameters. As shown in Figure 8b, the ports are allocated in the *Y*-direction, and the electric and magnetic fields are aligned in the *X*- and *Z*-directions, respectively. Figure 9 depicts the transmission coefficient while varying the slotted-strip line’s length. When the slotted-strip line length is with a value of Ls1 = 138 µm, the reflection coefficient at the center frequency of 0.309 THz is −23.70 dB, whereas if the length of the slotted is shortened to 110 μm, it exhibits dual resonances at 0.119 and 0.324 THz with transmission coefficients of −27.107 and −21.252 dB, respectively. The slotted-strip line displays dual resonance frequencies of 0.222 and 0.331 THz with a reflection coefficient of −31.11 and −16.42 dB, respectively, when its length is reduced to Ls3 = 88 μm. When the slotted-strip line length is decreased to Ls4 = 52 µm, the reflection coefficient at the 0.240 THz center frequency is −32.64 dB. Shortening the slotted-strip line to Ls5 = 30 µm demonstrates dual resonance frequencies of 0.172 and 0.334 THz, with corresponding transmission coefficients of −32.38 and −13.62 dB. When the length of the line is shortened to Ls6 = 25.5 µm, the slotted-strip line exhibits dual resonance frequencies of 0.182 and 0.336 THz with transmission coefficients of −33.91 and −11.23 dB, respectively. Table 4 summarizes the frequency and bandwidth effects of modifying the slotted split ring as the wave advances down the *Y*-axis.

## 6. Result, Analysis, and Discussion

In this section, the effective parameters of the proposed MTM unit cell are extracted using the postprocessing module of CST microwave studio, which employs the robust retrieval method [28,29], in conjunction with the knowledge of S21 and S11 to extract the parameters, and the resulting data is then analyzed. Additionally investigated are the characteristics of the electric field, magnetic field, and surface current for various resonances. In this section, a comparison of the proposed MTM with certain current efforts is conducted.

### 6.1. Electric Field, Magnetic Field, and Surface Current Analysis

**Figure 10:**
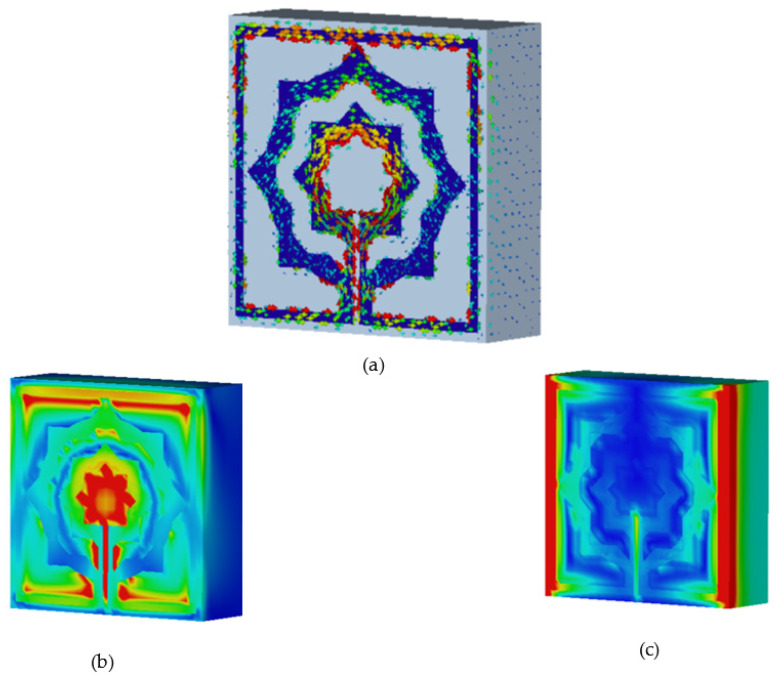
Analysis for: (**a**) current distribution; (**b**) magnetic field distribution; (**c**) electric field distribution for resonant frequency 0.25 THz.

The characteristics of a metamaterial will be comprehended with the aid of surface current, electric, and magnetic field studies. Using two waveguide ports to apply a plain wave with linear excitation enables the simulation of an incident wave at a great distance from the observed MTM item. In the direction of incidence, an open boundary condition is specified, and the input signal is a Gaussian pulse. An LC equivalent circuit is the cause of electromagnetic resonance, regardless of the geometrical configurations, according to the prevalent theory [30]. In addition, the inductance L is induced currents and a divide or gap represents capacitance C. As can be seen from Figure 10a, the current of the proposed metamaterial is concentrated on the upper side and the lower side of the design and it can also be noticed that the current concentrates on the inner part of the second ring and the tuning slot resulting from the excitation of capacitive effect [31], while lowing currents in these areas generate the magnetic field around them, as seen in Figure 10b. At this frequency, strong electric fields are observed in other sections of the resonator, as indicated in Figure 10c.

### 6.2. Analysis of Effective Parameters

**Figure 11:**
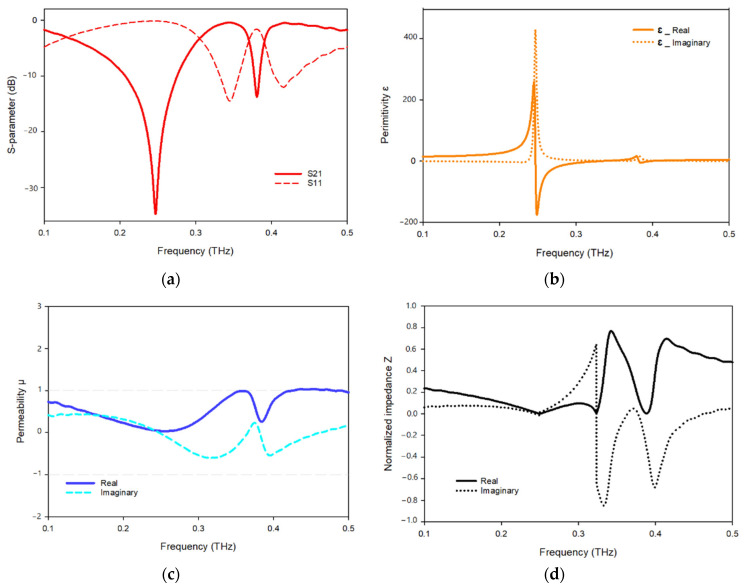
(**a**) S-parameter; (**b**) permittivity; (**c**) permeability; (**d**) normalized impedance.

The transmission and reflection coefficients, permittivity, permeability, and normalized impedance of the proposed metamaterial unit cell are shown in Figure 11, where the signal travels on the *Z*-axis, whereas the electric and magnetic fields are aligned in the x- and *y*-axes, respectively. As seen in Figure 11a, two resonances of S21 are followed by two resonances of S11, showing electrical resonance. As shown in Figure 11b, the permittivity plots have resonances at 0.249 Hz, while the permeability plots are close to zero in the subjected band. When the frequency of a wave approaches the frequency of resonance, the permeability of the material drops closer to zero. Figure 11c depicts a permeability plot with the lowest permeability values of 0.03 and 0.23, 0.26., respectively. In the region of negative permittivity, the real and imaginary components of the normalized impedance are positive, suggesting that the proposed MTM functions as a passive medium in these frequency ranges, as shown in Figure 11d.

**Figure 12:**
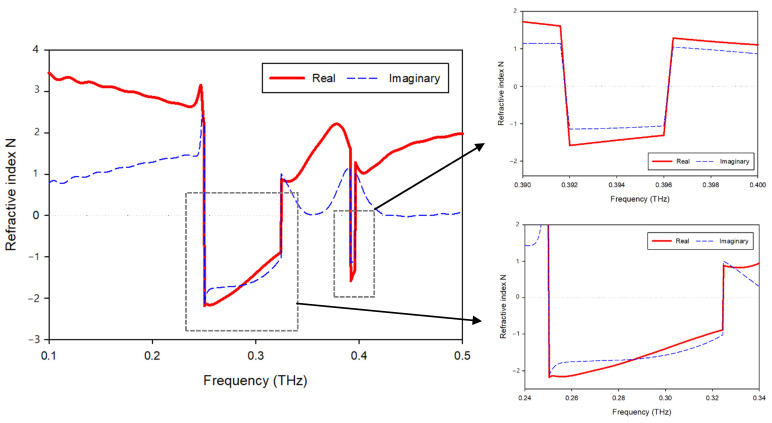
Refractive index of the proposed MMT.

Figure 12 displays the suggested unit cell structure’s real and imaginary refractive index. A negative refractive index occurs in two frequency bands, 0.250–0.324 and 0.392–0.396 THz. A misconception must be resolved: the metallic wire array exhibits negative permittivity, and the split-ring array exhibits negative permeability in the classical negative refractive index material [21,32]. The negative refractive index is the outcome of the double-negative (DNG) property. Moreover, other related articles regarding negative refractive index materials based on new structures also emphasize the double-negative feature, leading to the misconception that negative refractive index materials must have negative permittivity and negative permeability. A negative refractive index is achievable when the permittivity and permeability of a material fulfill the following formula [33]:

(4) $$\varepsilon_{r}\left|\beta \right|+\beta_{r}\left|\varepsilon \right|<0$$

where $\varepsilon_{r}$ and β*_r_* denote the real permittivity and permeability, respectively. It is evident that double-negative content meets this criterion. A negative refractive index also exists for the other metamaterials ($\varepsilon_{r}$ < 0, $\beta_{r}$ > 0 or $\varepsilon_{r}$ > 0, $\beta_{r}$ < 0) for which the condition is fulfilled [34]. The refractive index displayed in Figure 12 at the resonant frequencies demonstrates a shift from positive to negative. In the frequency range from 0.25 THz to 0.32 THz, negative refractive indices are recorded.

#### Analysis of Effective Parameters on the *Y*-Axis

**Figure 13:**
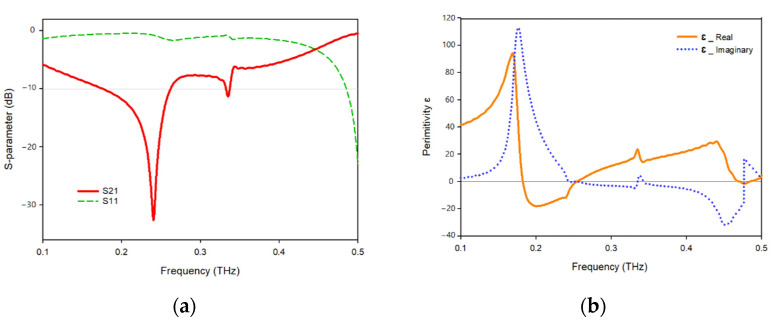

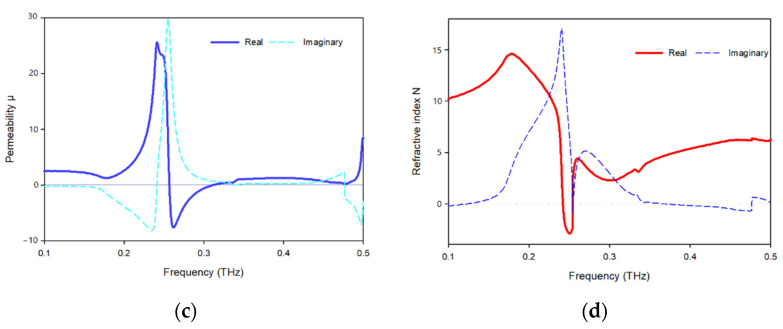
*Y*-axis effective parameter: (**a**) S-parameter; (**b**) permittivity; (**c**) permeability; (**d**) refractive index.

The transmission and reflection coefficients, permittivity, permeability, and refractive index of the design when it is positioned along the *Y*-axis are shown in Figure 13.

The metamaterial shows two resonant frequencies at 0.240 and 0.335 THz, as seen in Figure 13a, each frequency has a peak resonance of −32.64 dB and −11.32 and ranges between 0.176–0.263 and 0.332–0.337 THz dB, respectively. In Figure 13b, the real part of the permittivity shows negative values between 0.183–0.255 while permittivity begins to become positive as permeability begins to decrease, as Figure 13c shows the permeability attains a negative value in the frequency range 0.257–0.312 THz. In Figure 13d, the refractive index shows negative values up to −3 for frequencies ranging between 0.242–0.254 THz.

### 6.3. Analysis of the Array Structure

**Figure 14:**
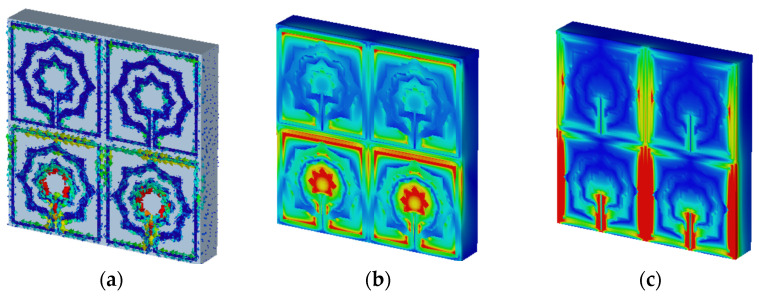
Analysis for 2 × 2 array: (**a**) current distribution; (**b**) magnetic field distribution; (**c**) electric field distribution for resonant frequency 0.25 THz.

**Figure 15:**
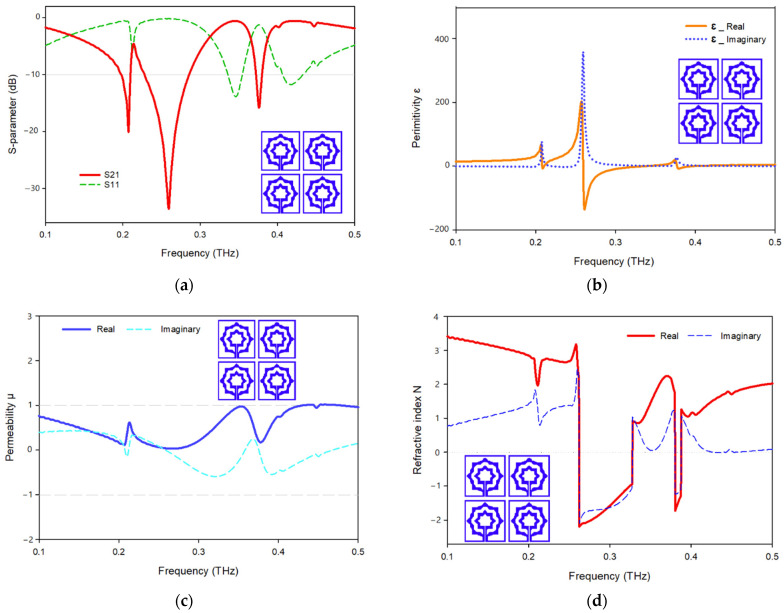
Effective parameters of the 2 × 2 array structure: (**a**) scattering parameters; (**b**) permittivity; (**c**) permeability; (**d**) refractive index.

The unit cell array structure’s performance is examined and discussed in this section. Figure 11 shows the 2 × 2 array structure’s effective parameters. With the help of surface current, electric, and magnetic field research, the properties of a metamaterial will be realized. In the suggested 2 × 2 array structure, the majority of the current is focused on the bottom cells, where it flows strongly in the center of the rings toward the slotted-strip line, as well as in the lower and upper sides of the outer frame. In contrast to the top cells, the majority of the current is distributed on the outer ring’s upper side as can be seen in Figure 14a, while the magnetic field surrounding these places is generated by the movement of currents related to that seen in Figure 14b. The electric field distribution shown in Figure 14c indicates that a significant electric field occurs on the left and right sides and at the slotted-strip line of each cell, all of which contribute to the fluctuation in capacitance. As shown in Figure 15a, the frequency of the 2 × 2 structure ranges from 0.228 to 0.286 THz; it has resonant frequencies of 0.259 THz and transmission coefficients of −33.6 dB, the second resonant frequency is at 0.376 THz, and it ranges from 0.372 to 0.380, having a transmission coefficient of −15.8 dB. Additionally, another resonant frequency is located at 0.207 THz, having a transmission coefficient of −20.1 dB and ranges from 0.198 to 0.210 THz. The real and imaginary effective permittivities of the 2 × 2 structure are shown in Figure 15b. Between 0.259 and 0.335 THz and 0.377 and 0.397 THz, permittivity shows negative frequency ranges, whereas permeability has near-zero values. As can be seen in Figure 15c, the 2 × 2 structure has both a real and imaginary permeability. Permeability is near zero and has three resonance frequencies at 0.207 THz, the lowest value for 0.267 THz is 0.03, and the highest value for 0.378 THz is 0.17. The 2 × 2 array’s real and imaginary refractive indices are shown in Figure 15d. The ranges of 0.262–0.378 THz and 0.380–0.388 THz are the frequency ranges for the negative refractive index.

**Figure 16:**
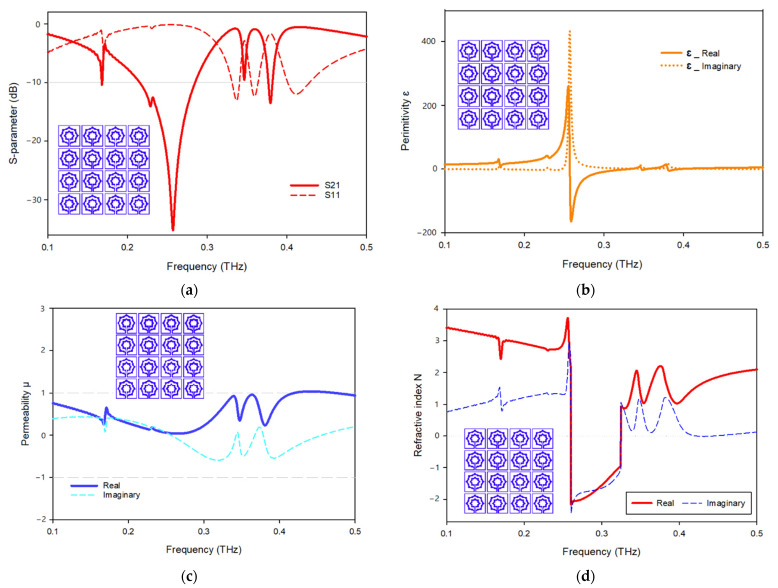
Effective parameters of the 4 × 4 array structure: (**a**) scattering parameters; (**b**) permittivity; (**c**) permeability; (**d**) refractive index.

Figure 16 demonstrates the effective parameters of the 4 × 4 array structure. Figure 16a indicates that it has a frequency range of 0.257–0.379 and 0.376–0.381 THz with resonant frequencies of 0.257 and 0.379 THz and transmission coefficients of −35.2 and −13.5 dB, respectively. Figure 16b depicts the real and imaginary effective permittivity of the 4 × 4 structure. Permittivity plots exhibit a negative frequency range between 0.257 and 0.332, 0.347 and 0.355, and 0.380 and 0.397 THz, whereas permeability plots are near zero in the relevant band. However, Figure 16c depicts the real and imaginary permeability of the 4 × 4 structure. The real permeability is close to zero and has resonant frequencies at 0.168, 0.346, 0.265, and 0.380 THz; the lowest value for 0.265 THz is 0.03, and the highest value for 0.346 THz is 0.36. Figure 16d depicts the real and imaginary refractive index of the 4 × 4 array configuration. The frequency ranges for negative refractive index are indicated in the frequency span of 0.26–0.324 THz.

**Figure 17:**
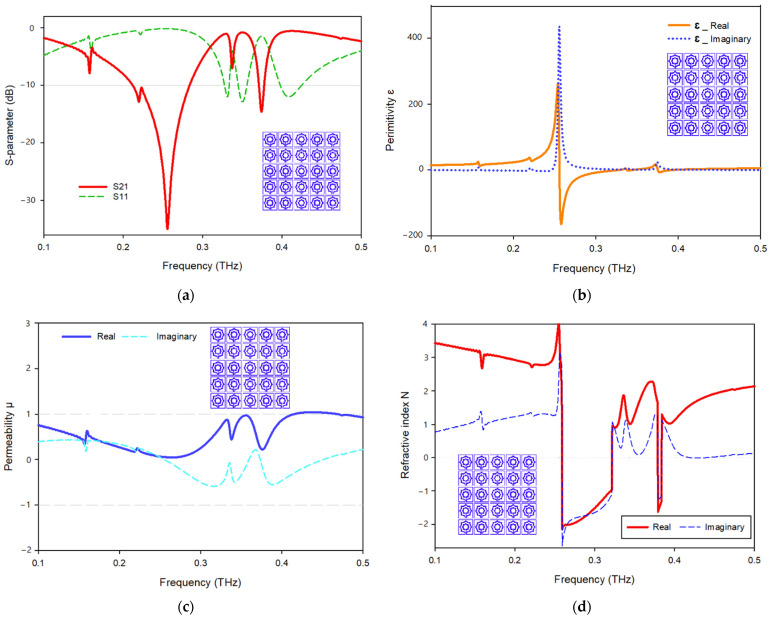
Effective parameters of the 5 × 5 array structure: (**a**) scattering parameters; (**b**) permittivity; (**c**) permeability; (**d**) refractive index.

The effective characteristics of the 5 × 5 array structure are shown in Figure 17. It has a frequency range of 0.212–0.284 and 0.371–0.371 THz, with resonant frequencies of 0.256 and 0.374 THz and transmission coefficients of −35.02 and −14.60, respectively, as shown in Figure 17a. Figure 17b illustrates the effective real and imaginary permittivity of the 5 × 5 structure. The permittivity plots display a negative frequency range between 0.260 and 0.329, 0.338 and 0.345, and 0.375 and 0.394 THz, whereas permeability plots are close to zero in the relevant region. Figure 17c illustrates the real and imaginary permeability of the 5 × 5 construction. The permeability is close to zero at frequencies of 0.157, 0.267, 0.338, and 0.376 THz, with the lowest value being 0.04 for 0.267 THz and the highest value being 0.44 for 0.338 THz. The real and imaginary refractive indices of the 5 × 5 array layout are shown in Figure 17d. The negative refractive index has dual frequency ranges of 0.259–0.322 and 0.379–0.384 THz. In both array structure investigations, it is evident that the available frequency ranges for the effective parameters are almost identical to those of the single unit cell, except for a few frequency bands that altered significantly and created minimal distortion owing to coupling effects.

The proposed MTM is presented to compare and summarize the proposed MTM with some recently published articles in state of the art, as shown in Table 5, whereas the size, resonant frequency, bandwidth, dielectric substrate, metallic layer, the ability to be tuned, and the material characteristics such as ENG or DNG are considered as the most critical parameters.

## 7. Conclusions

This paper presents a compact and tunable metamaterial consisting of a metallic star-shaped split-ring resonator with a tunable gap in the center to be utilized in 6G applications. This proposed metamaterial provides two resonances at 0.248 and 0.383 THz with an operating frequency range of 0.207–0.277 and 0.382–0.390 THz, respectively, whereas the overall size is 160 × 160 μm^2^. Mutual coupling between the array elements is decreased due to the MTM’s symmetric structure, and the array shows a similar transmission coefficient (S21) response to the unit cell for *Z* and *Y* principal axis wave propagation. Furthermore, the equivalent circuit of the proposed MTM is modeled in ADS and verified by contrasting the closely related S21 response with CST. The MTM characteristics show double-negative index, negative permittivity, and near-zero permeability have also been analyzed. Through the analysis of the electric field, magnetic field, and surface current, the contribution of the various components of the MTM unit cell to resonance is investigated. Due to its low profile size with high effective DNG, negative ENG, and near-zero permeability characteristics, the proposed MTM can be utilized with various wireless devices in THz and 6G applications, especially to enhance the gain and directivity of the antenna. Upcoming work is foreseen to utilize the proposed metamaterial in a 6G massive multiple-input multiple-output (MIMO) antenna array design to improve the overall performance. 

## Figures and Tables

**Table 1:** Design parameters of proposed MTM unit cell.

Parameter	Dimensions (μm)	Parameter	Dimensions (μm)
L	160	W1	4
L1	150	W2	10
L2	140	R1	57
L3	96	R2	50
L4	56	Ts	50
Ls	52	T_M_	0.4

**Table 2:** The transmission coefficient (S21) of sequential unit cell steps.

Substructure	Resonance Frequency (THz)	Bandwidth (THz)	Resonance Peak (dB)
Design 1	0.157	0.144–0.168	−29.33
Design 2	0.194, 0.373	0.169–0.214, 0.367–0.379	−34.12, −17.54
Design 3	0.203, 0.375	0.176–0.223, 0.368–0.382	−34.31, −18.23
Proposed unit cell	0.248, 0.383	0.207–0.277, 0.380–0.385	−35.72, −12.77

**Table 3:** Performance comparison of the proposed MTM for different slotted-strip line lengths.

Ls (µm)	Frequency (THz)	Bandwidth (THz)	Ls (µm)	Frequency (THz)	Bandwidth (THz)
138	0.298	0.260–0.327	52	0.248, 0.383	(0.207–0.277), (0.382–0.390)
110	0.319, 0.145	(0.281–0.344), (0.138–0.150)	30	0.192, 0.362	(0.170–0.207), (0.348–0.373)
88	0.239, 0.360	(0.203–0.262), (0.352–0.370)	25.5	0.201, 0.374	(0.175–0.220), (0.365–0.380)

**Table 4:** Performance comparison of MTM for different slotted-strip line lengths in the *Y*-direction.

Ls (µm)	Frequency (THz)	Resonance Peak (dB)	Bandwidth (THz)
138	0.309	−23.70	(0.263–0.317)
110	0.119, 0.324	−27.107, −21.252	(0.109–0.126), (0.265–0.329)
88	0.222, 0.331	−31.11, −16.42	(0.172–0.240), (0.315–0.334)
52	0.240	−32.64	(0.176–0.262)
30	0.172, 0.334	−32.38, −13.62	(0.141–0.190), (0.237–0.337)
25.5	0.182, 0.336	−33.91, −11.23	(0.145–0.205), (0.333–0.338)

**Table 5:** Proposed MTM comparison with the reported state of the art.

Ref.	Size (µm^2^)	Fre. (THz)	BW (THz)	Substrate	Metallic Layer	No. of Axes	ENG (THz)	Near-Zero Mu	DNG (THz)	Tunable
[20]	90 × 90	0.330, 0.650	(0.298–0.388) (0.652–0.780)	GaAs	Titanium, platinum and gold	1	0.298–0.388 and 0.652–0.780	-	no	no
[23]	200 × 200	0.37	0.340–0.400	GaAs	-	1	no	-	no	no
[24]	180 × 212	1.08	(1.053–1.140)	Quartz	Silver	1	-	-	1.04–1.09	no
[35]	55 × 55	0.90	(0.800–1.800)	SiO2	-	1	no	-	no	no
[36]	64 × 64	0.6, 1.1	-	RT5880	Gold	1	-	-	yes	no
This work	160 × 160	0.25, 0.386	(0.210–0.28), (0.382–0.390)	Polyimide	Copper	2	0.25–0.33 and 0.39–0.41	0.1–0.5	0.25–0.32 and 0.391–0.396	yes

## Data Availability

Not applicable.
